# Octreotide inhibits secretion of IGF-1 from orbital fibroblasts in patients with thyroid-associated ophthalmopathy via inhibition of the NF-κB pathway

**DOI:** 10.1371/journal.pone.0249988

**Published:** 2021-04-22

**Authors:** Sung Eun Kim, Jia Kim, Ji-Young Lee, Seong-Beom Lee, Ji-Sun Paik, Suk-Woo Yang

**Affiliations:** 1 Department of Ophthalmology, Seoul St. Mary’s Hospital, College of Medicine, The Catholic University of Korea, Seoul, Korea; 2 Department of Pathology, College of Medicine, The Catholic University of Korea, Seoul, Korea; 3 Department of Ophthalmology, Yeouido St. Mary’s Hospital, College of Medicine, The Catholic University of Korea, Seoul, Korea; Indian Agricultural Research Institute, INDIA

## Abstract

**Purpose:**

We investigated the effect of octreotide, a long-acting somatostatin (SST) analogue, on IGF-1 secretion and its possible mechanism of action in orbital fibroblasts (OFs) from patients with thyroid-associated ophthalmopathy (TAO).

**Materials and methods:**

OFs were isolated from the orbital fat of patients with TAO or healthy individuals. The expression level of insulin-like growth factor (IGF)-1, at the protein and mRNA level, was determined with ELISA and quantitative RT-PCR, respectively. The expression pattern of somatostatin receptor (SSTR) 2, which has the highest affinity for octreotide, was examined by flow cytometry. The activity of NF-κB pathway was determined by examining the levels of phosphorylation of IKKα/β and p65, and degradation of IκB via western blot analysis, and by measuring the activity of NF-kB-dependent luciferase via transfection with plasmids containing luciferase and NF-κB binding site.

**Results:**

OFs from patients with TAO showed significantly higher levels of IGF-1 secretion and NF-κB activity even in the absence of stimulation, compared to those from controls. Treatment with octreotide reduced the level of IGF-1 secretion in OFs from patients with TAO, but not in OFs from controls. OFs from patients with TAO expressed higher levels of SSTR2 on the cell surface, compared to controls. In addition, the expression of IGF-1 at the protein and mRNA level was dependent on the activity of NF-κB pathway in OFs from patients with TAO. Furthermore, treatment with octreotide reduced on the activity of NF-κB pathway in OFs from patients with TAO.

**Conclusion:**

OFs from patients with TAO showed significantly higher levels of IGF-1 secretion via up-regulation of NF-κB activity. Treatment with octreotide inhibited the secretion of IGF-1 by reducing the NF-κB pathway in OFs, which expressed higher levels of SSRT2 on the cell surface, from patients with TAO.

## Introduction

Thyroid-associated ophthalmopathy (TAO) is an autoimmune disease of the orbit involving infiltration of inflammatory cells and proliferation of orbital fibroblasts (OFs) leading to accumulation of the extracellular matrix (ECM) and hypertrophy of extraocular muscles and adipose tissue [1]. These pathological changes contribute to the characteristic manifestations of TAO, including eyelid retraction and edema, exophthalmos, limitation of ocular movement, and even vision impairment caused by compressive optic neuropathy or corneal breakdown [2].

OFs play key role in the development of TAO because they express thyroid stimulating hormone receptor (TSHR), which is targeted by autoantibodies, thyroid-stimulating immunoglobulin (TSI), thus triggering inflammation [3]. In addition to TSI, insulin-like growth factor-1 (IGF-1) is also an important player in the development of TAO. IGF-1 is a polypeptide that is involved in the growth, differentiation, and metabolism of various cells [4]. Local production of IGF-1 by OFs is implicated in the growth of OFs, in an autocrine or paracrine manner, subsequently leading to the development of TAO [5]. In addition, IGF-1 has been reported to enhance the effect of TSH and TSI on TSHR signaling not only in thyroid cells but also in extra-thyroid cells, including OFs [6].

Octreotide is a synthetic octapeptide that pharmacologically mimics natural somatostatin (SST). It has the highest affinity for the SST receptor (SSTR) 2 compared to other SSTR subtypes [7]. There are five SSTR types with varying affinities for octreotide. SSTR2 shows the highest affinity for octreotide, whereas SSTR3 shows intermediate affinity and SSTR5 shows moderate affinity. SSTR1 and SSTR4 do not bind to octreotide [7]. In addition, the expression levels of SSTR1 and SSTR2 are increased in OFs and lymphocytes from orbital tissues of patients with TAO, compared to control individuals [8,9]. Octreotide, a long-acting SST analog, is known to decrease the secretion of growth hormone (GH) and IGF-1 in patients with acromegaly [10]. Thus, octreotide is used for the treatment of GH/IGF-1-related diseases, such as acromegaly, TSH-secreting pituitary adenomas, and gastro-entero and pancreatic neuroendocrine tumor [11–13]. Treatment with 1,000 nM octreotide could neutralize the increase in *IGF-1* mRNA level and significantly reduce the proliferation of cultured OFs by 75% in patients with TAO [5].

From mid-1990s to mid-2000s, many researchers and physicians have conducted clinical studies to determine whether octreotide is effective in treating TAO. Initial studies in a small number of patients have reported that treatment with octreotide improved the clinical symptoms of patients with TAO [14,15]. However, clinical trials conducted in mid-2000s with more patients reported that, overall, octreotide did not significantly ameliorate TAO [16,17], but interestingly, it improved some of the symptoms, such as lid retraction [18] and proptosis [17]. Thus, octreotide may help to ameliorate the symptoms of patients with TAO who have high levels of SSTR2 in orbital tissues. It is, therefore, reasonable to investigate how octreotide inhibits secretion of IGF-1 from OFs of patients with TAO.

Here, we initially compared the levels of IGF-1 secretion between OFs from control individuals and patients with TAO. We also measured the level of SSTR2 expression, because SSTR2 is expressed in OFs and has the highest affinity for octreotide compared to other SSTR subtypes [7]. We then investigated the effect of octreotide on IGF-1 secretion in OFs and its underlying mechanism. Our results suggest that the NF-κB pathway is responsible for the maintenance of increased IGF-1 secretion. Although multiple signaling pathways are involved in the regulation of IGF-1 expression, we postulate that the NF-κB pathway is a major signaling pathway that regulates IGF-1 expression since previous evidence suggests that the NF-κB pathway is implicated in the expression of IGF-1 [19].

## Materials and methods

### Reagents and antibodies

Octreotide was purchased from Sigma-Aldrich Co. Ltd. (St. Louis, MO, USA). Bay 11–7085, which is an inhibitor of NF-κB, was purchased from Calbiochem (La Jolla, CA, USA). Octreotide was dissolved in H_2_O. Bay 11–7085 was dissolved in 0.1% dimethyl sulfoxide (DMSO). Control medium contained only 0.1% DMSO. The antibody against SSTR2 (product PA3-16736) was purchased from Thermo Fisher Scientific (Waltham, MA, USA). The rabbit IgG isotype control was purchased from BD Pharmingen (San Jose, CA, USA). Antibodies against phospho-IKKα/β, IKKβ, phospho-NF-kB p65, and NF-kB p65 were purchased from Cell Signaling Technology (Beverly, MA, USA). Antibodies against IκBα and β-actin were purchased from Santa Cruz Biotechnology (Santa Cruz, CA). Horseradish peroxidase-conjugated secondary antibodies were purchased from Merck Millipore (Burlington, MA, USA). Alexa Fluor-647-conjugated goat anti-rabbit antibody was purchased from Cell Signaling Technology.

### Cell cultures

Human OFs were obtained from orbital fat obtained from decompression surgery in patients with TAO (n = 7) or from upper lid blepharoplasties in patients with no prior history of inflammatory/immune or thyroid disease (n = 8). Orbital fat explants were minced in small pieces, placed in plastic culture dishes, and covered with Dulbecco’s Modified Eagle’s medium (GIBCO BRL, Grand Island, NY) supplemented with 20 mM HEPES (Fisher Scientific, Atlanta, GA), 10% fetal bovine serum (FBS, GIBCO BRL), 100 U/ml penicillin, and 100 μg/ml streptomycin (Bio Whittaker Inc., Walkersville, MD). Cultures were maintained at 37°C in a 5% CO_2_ humidified incubator until the fibroblasts reached 70% confluence. Non-adherent cells and fat tissues were then removed, and the established fibroblasts were passaged with gentle trypsin/EDTA treatment. Fibroblasts were not used beyond passage 10 from the initial culture. Institutional Review Board/Ethics Committee of Seoul St. Mary’s Hospital approved this research (KC10TISE0743). Informed written consent was obtained from the donors prior to OF isolation, according to guidelines from the Institutional Review Board of Seoul St. Mary’s Hospital. The study was conducted in accordance with the tenets of the Declaration of Helsinki.

### Quantification of IGF-1

The culture medium of OFs was analyzed for human IGF-1 content using a standard sandwich enzyme linked immunosorbent assay (ELISA) kit (R&D Systems, Minneapolis, MN) according to manufacturer’s instructions.

### Flow cytometry

The expression level of cell surface SSTR2 was examined by flow cytometry. Human OFs were trypsinized with 0.05% trypsin-EDTA (GIBCO BRL) and then resuspended in complete media. Cells were centrifuged at 1,000 rpm for 5 min and then fixed with 2% paraformaldehyde for 15 min at room temperature (RT). Cells were suspended in fluorescence-activated cell sorting (FACS) buffer (0.1% NaN3 + 0.5% BSA in PBS) and then incubated with the primary antibody at RT for 1 h. Rabbit IgG isotype was used as a negative control. After two washes with FACS buffer, cells were incubated with the secondary Alexa Fluor-647-conjugated antibody at RT for 30 min. Cells were then washed twice with FACS buffer, resuspended in FACS buffer, and analyzed with flow cytometry (FACS Canto II, BD Bioscience, San Jose, CA).

### Cell viability assay

Cell viability was evaluated using the 3-(4,5-dimethylthiazol-2-yl)-2,5-diphenyltetrazolium bromide (MTT) reduction assay in 96-well plates. Cells were treated based on the experimental protocols and 10 μl of a 5 mg/ml MTT solution was then added to each well. After incubation in a 5% CO_2_ incubator for 2 h at 37°C, media were removed and 100 μl HCl-isopropyl alcohol were added to each well. After incubation for 10 min, 100 μl distilled water were added to each well. The optical density for each well was then measured at 570 nm in a spectrometer (μ-Quant, BioTek instruments, Inc., Winooski, VT).

### Western blot analysis

Human OFs were removed from the incubator and placed on ice. Cells were then washed three times with ice-cold PBS and lysed for 30 min with RIPA lysis buffer [50 mM Tris-HCl (pH 7.4), 1% Triton X-100, 150 mM NaCl, 0.1% sodium dodecyl sulfate (SDS), 0.5% sodium deoxycholate, 100 mM phenylmethylsulfonyl fluoride, 1 μg/ml leupeptin, 1 mM Na_3_VO_4_, and 1X Complete^™^ Protease Inhibitor Cocktail (Santa Cruz Biotechnology)]. Equal amounts of protein extracts were loaded onto 10–15% SDS-PAGE gels, electrophoresed, and transferred to PVDF membranes (Millipore, Bedford, MA). Membranes were blocked in Tris-buffered saline with 0.05% Tween-20 (TBST) supplemented with 5% powdered milk or 5% bovine serum albumin, and then incubated with a primary antibody against the designated protein. Blots were then washed with TBST and incubated with a horseradish peroxidase-conjugated secondary antibody in TBST with 5% powdered milk. Bound antibodies were detected with the Amersham ECL Prime Western Blotting Detection kit (GE Healthcare, Buckinghamshire, UK).

### Assay for NF-kB-dependent promoter activity

pGL4.32[luc2P/NF-κB-RE/Hygro] and pRL-TK plasmids were purchased from Promega (Madison, WI, USA). OFs were plated at a density of 5 x 10^4^ cells/well in a 24-well plate and were co-transfected with pGL4.32[luc2P/NF-κB-RE/Hygro] and pRL-TK, using the Viromer^®^ RED transfection reagent (Lipocalyx, Halle, Germany) according to the manufacturer’s instructions. After 24 h, cells were harvested for the luciferase assay. Firefly and renilla luciferase activities were measured using a TransDetect^®^ Double-Luciferase Reporter Assay Kit (TransGen Biotech, Haidian District, Beijing, China) in a SpectraMax L Microplate Luminometer (Molecular Devices, Sunnyvale, CA, USA). The results are expressed as the ratio of firefly to renilla luciferase activity (Fluc/Rluc).

### Quantitative real time reverse transcription-polymerase chain reaction (RT-PCR)

*IGF-1* mRNA expression was determined with quantitative real-time RT–PCR. Total RNA was extracted using the Relia Prep^™^ RNA Cell Miniprep System kit (Promega, Madison, WI, USA) and converted to cDNA using the Transcriptor First Strand cDNA Synthesis kit (Roche Diagnostics, Mannheim, Germany), according to the manufacturer’s instructions. To quantify *IGF-1* mRNA, quantitative real time RT–PCR was performed using the iQ^™^ SYBR^®^ Supermix kit (Bio-Rad Laboratories, Hercules, CA) in a CFX Connect^™^ real-time system (Bio-Rad Laboratories, Hercules, CA). RT-PCR was performed in triplicates at 95°C for 2 min followed by 40 cycles of amplification (95°C for 10 s, 61°C for 30 s, and 72°C for 15 s). The relative amount of *IGF-1* mRNA was determined by subtracting *IGF-1* cycle threshold (Ct) values from the Ct values for *28S* rRNA. The following primers for *IGF-1* and *28S* rRNA were used: For *IGF-1*, forward primer (5’-TGG ATG CTC TTC AGT TCG TG-3’) and reverse primer (5’-CTG ACT TGG CAG GCT TGA G-3’). For *28S* rRNA, forward primer (5’-ACG GTA ACG CAG GTG TCC TA-3’) and reverse primer (5’-CCG CTT TCA CGG TCT GTA TT-3’).

### Statistical analysis

All results are expressed as mean ± SD from at least three independent experiments. Statistical significance was determined via the student’s t test for two points; *p*-value < 0.01 or *p*-value < 0.05 was considered to be statistically significant.

## Results

### Characteristics of patients with TAO

The study included 7 patients with TAO and 8 controls. Three males and four females with mean age 55.3 years old (range 23–73) in the TAO group and four males and four females with mean age 60 years old (range 22–75) in the control group were included. All TAO patients had been diagnosed with Graves’ disease. Among them, seven patients had been treated with methimazole and one patient had thyroidectomy. In TAO group, seven patients had previously undergone prednisolone therapy and one patient had undergone radiation therapy in the acute phase of the disease. The clinical activity score ranged from 1 to 3. At the time of orbital decompression, they had experienced at least six months of inactive disease status with a euthyroid hormonal state. The characteristics of patients with TAO are described in Table 1. The control group had no inflammatory/immune diseases or thyroid disease. In the control group, the orbital fat was obtained from twelve patients who had upper eyelid blepharoplasties.

**Table 1:** Characteristics of patient with thyroid-associated ophthalmopathy from whom orbital fibroblasts were obtained.

TAO Patient number	Age	Sex	Smoking	Grave’s disease	Grave’s disease treatment			TAO treatment			Euthyroid state	TSH receptor antibody	CAS
					Radioactive Iodine	Methimazole	Thyroidectomy	Surgery	Prednisolone	Radiation			
**#103**	23	F	No	Yes	No	Yes	No	Yes	No	No	Yes	+	2
**#107**	50	F	No	Yes	No	Yes	No	Yes	Yes	Yes	Yes	+	3
**#110**	65	M	No	Yes	No	No	Yes	Yes	Yes	No	Yes	+	1
**#120**	56	M	Yes	Yes	No	Yes	No	Yes	Yes	No	Yes	+	3
**#135**	61	M	No	Yes	No	Yes	No	Yes	Yes	No	Yes	+	1
**#137**	59	F	No	Yes	No	Yes	No	Yes	Yes	No	Yes	+	3
**#162**	73	F	No	Yes	No	Yes	No	Yes	Yes	No	Yes	+	3

All patients with TAO had experienced at least 6 months of inactive disease status with a euthyroid hormonal condition.

Non-TAO patients; #71 (female, 61 years), #111 (female, 68 years), #116 (male, 60 years), #136 (male, 59 years), #150 (female, 65 years), #152 (female, 22 years), #155 (male, 70 years), #158 (male, 75 years).

CAS, clinical activity score; TSH, thyroid-stimulating hormone.

### OFs from patients with TAO secreted significantly higher levels of IGF-1

**Fig 1:**
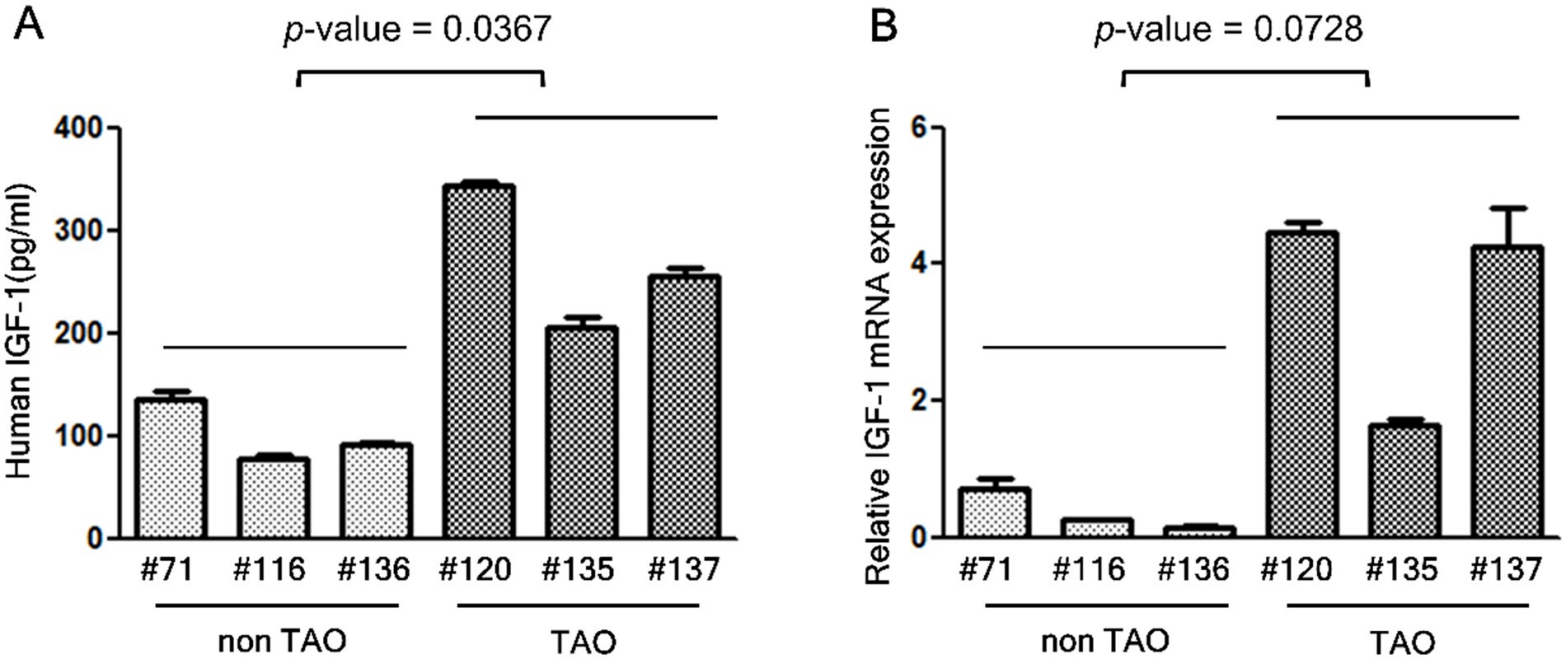
Orbital fibroblasts (OFs) from TAO patients secreted higher levels of IGF-1 and expressed higher levels of *IGF-1* mRNA. (A) OFs from control individuals (#71, #116, #136) and patients with TAO (#120, #135, #137) were plated at a concentration of 2 x 10^5^ cells/well in a 6-well plate. After 24 hours, the culture medium was analyzed for IGF-1 content using an ELISA kit (R&D Systems, Minneapolis, MN). (B) *IGF-1* mRNA expression was determined by quantitative real time-RT–PCR.

We initially examined the basal levels of IGF-1 secretion in non-treated OFs from controls and patients with TAO. As shown in Fig 1, OFs from patients with TAO spontaneously secreted higher levels of IGF-1 (*p*-value = 0.0367) and expression of *IGF-1* mRNA (*p*-value = 0.0728) even in the absence of stimulation, compared to control. This finding indicates that OFs in TAO patients still have the ability to express higher level of IGF-1 after separation from orbital tissues.

### Treatment with octreotide reduced the increased level of IGF-1 secretion in OFs from patients with TAO

**Fig 2:**
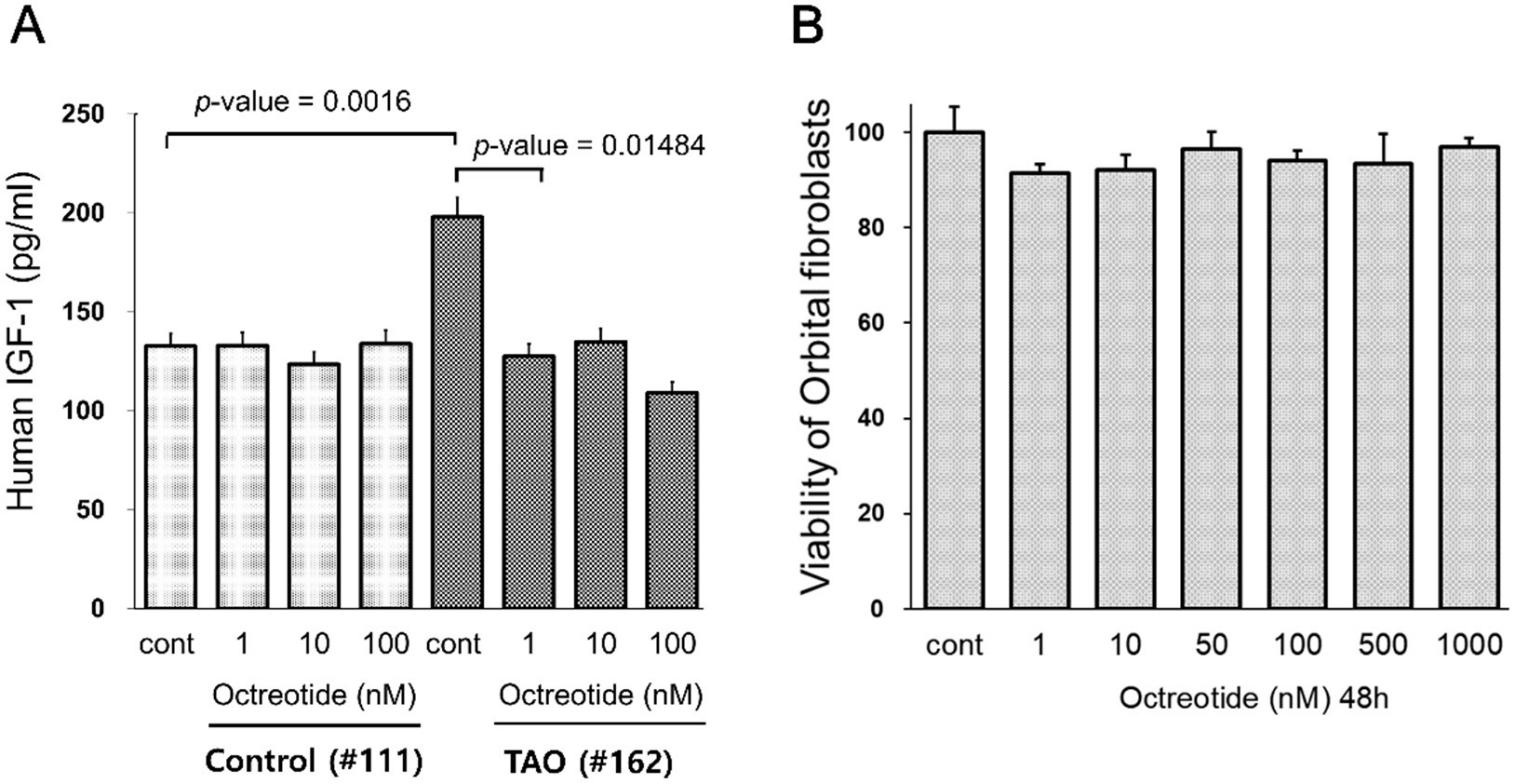
Treatment with octreotide reduced the levels of IGF-1 secretion in OFs from patients with TAO. (A) OFs were plated at a concentration of 2 x 10^5^ cells/well in a 6-well plate. After 24 hours, cells were treated with octreotide at the indicated concentration for 24 hours. The culture medium from one control individual (#111) and one patient with TAO (#162) was analyzed for IGF-1 content with ELISA. (B) OFs, which were derived from a control (#111), were treated with octreotide at the indicated concentrations for 48 hours. Cell viability was determined using an MTT assay.

We then treated OFs with octreotide to examine its effect on IGF-1 secretion. Treatment with octreotide at concentrations of 1–100 nM resulted in a significant reduction of IGF-1 secretion to the control level in one TAO patient (#162). Octreotide reduced the level of IGF-1 secretion by about 50% at a concentration of 100 nM (Fig 2A). However, treatment with octreotide did not induce any effect on IGF-1 secretion in OFs from one control individual (#111).

In addition, we examined the cytotoxicity of octreotide on OFs. Cells, which were derived from a control (#111) were treated with the indicated concentrations of octreotide for 48 h. Treatment with octreotide at concentrations up to 1,000 nM had no effect on OF viability (Fig 2B).

### OFs from patients with TAO expressed higher levels of somatostatin receptor (SSTR) 2

**Fig 3:**
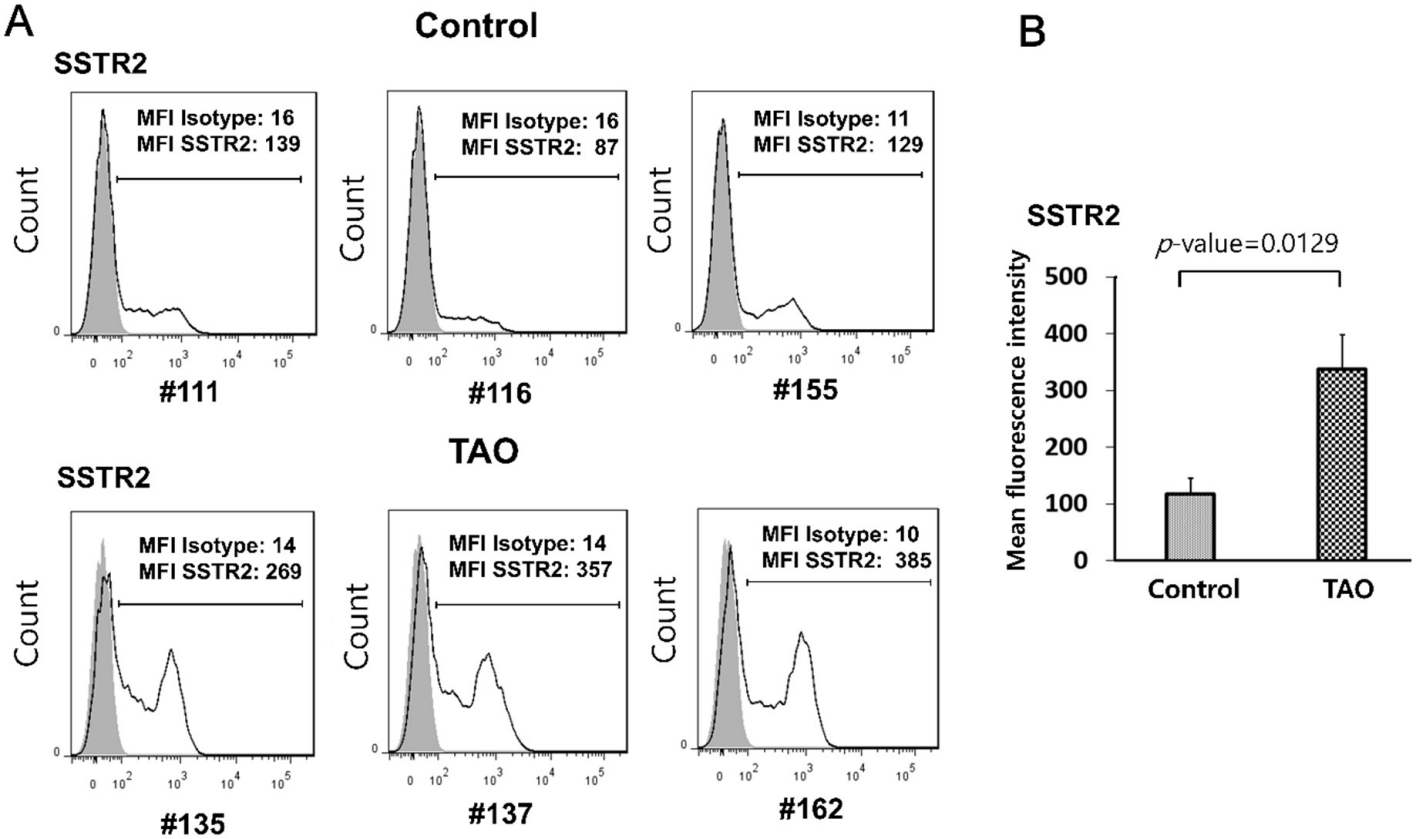
OFs from patients with TAO expressed higher levels of somatostatin receptor type (SSTR) 2. (A) OFs were plated at a concentration of 2 x 10^5^ cells/well in a 6-well plate. After 24 hours, the levels of SSTR2 on the cell surface of OFs from control individuals (#111, #116, #155) and patients with TAO (#135, #137, #162) were examined by flow cytometry. The gray open histogram represents staining with isotype control antibodies. (B) Mean fluorescence intensity of SSTR2 was 2.85-fold higher in patients with TAO compared to control individuals.

Radiolabeled octreotide shows a predominant affinity for SSTR2, compared to other SSTR subtypes [7]. *SSTR2* mRNA is detected in control individuals and in patients with TAO [20]. Thus, we investigated the level of SSTR2 in OFs from the two groups. As shown in Fig 3, OFs from patients with TAO (#135, #137, #162) showed significantly higher levels of SSTR2 expression on their cell surface, compared to controls (#111, #116, #155) [mean fluorescence intensity (MFI) by flow cytometry: 269, 357 and 385 in TAO and 138, 87 and 129 in control]. These results suggest that the difference in the degree of response to octreotide in OFs between patients with TAO and control individuals (Fig 2) is due to the difference in the expression of SSTR2.

### OFs from patients with TAO showed significantly higher level of NF-κB activity

Next, we designed an experiment to elucidate the underlying mechanism involved in the upregulation of IGF-1 expression in OFs in TAO patients. Although IGF-1 activates the NF-κB pathway [21], the NF-κB pathway may also induce expression of IGF-1 [19]. Thus, we focused on the role of the NF-κB pathway in the expression of IGF-1 from OFs.

In stimulated cells, IKKα/β is activated and phosphorylated (p)-IKKα/β then induces phosphorylation of IκB. IκB phosphorylation leads to IκB polyubiquitination and its subsequent degradation by 26S proteasomes. The NF-κB proteins are released from IκB and translocated to the nucleus, where they bind to the promoter regions of NF-κB-responsive genes.

**Fig 4:**
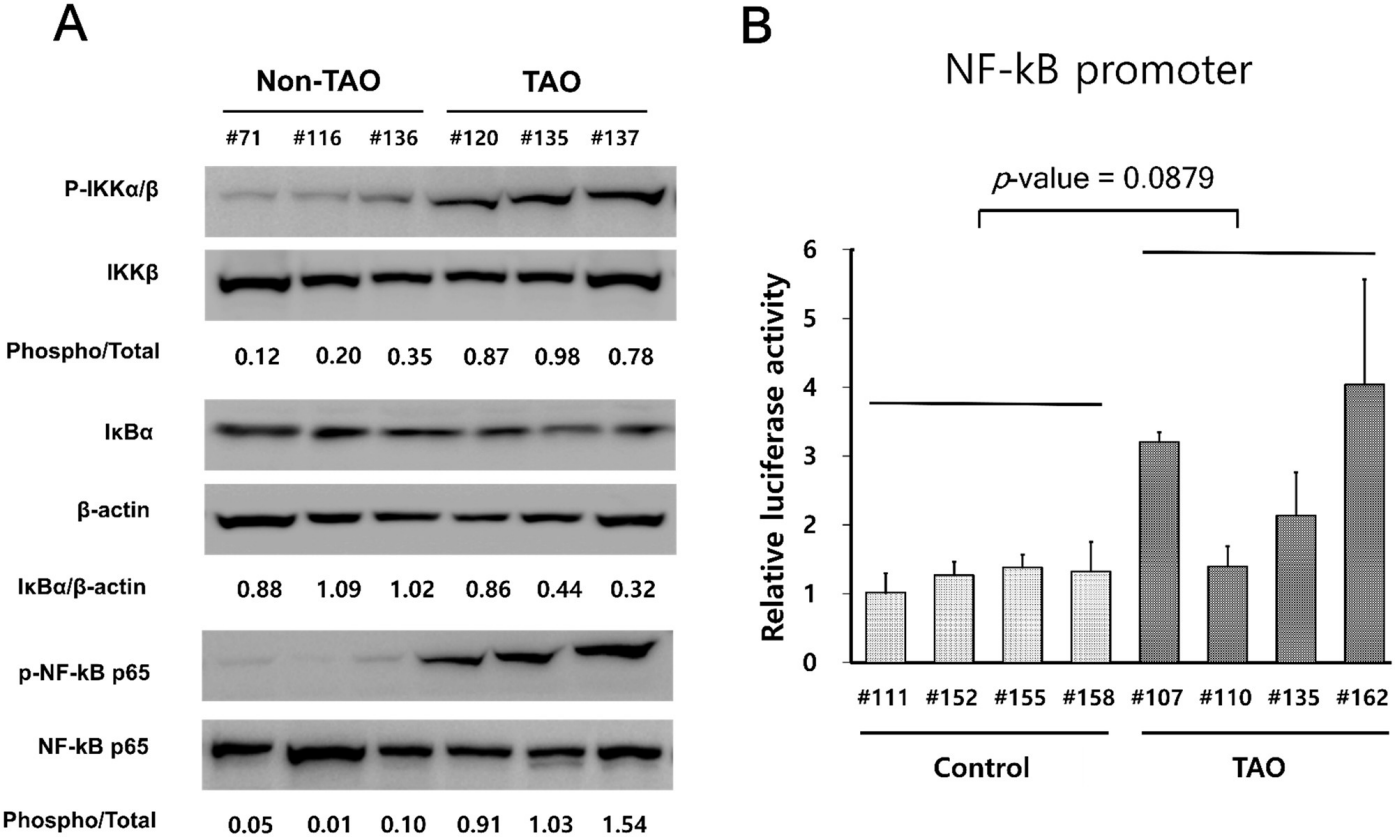
OFs from patients with TAO showed higher levels of NF-κB activity. (A) OFs were plated at a concentration of 2 x 10^5^ cells/well in a 6-well plate. After 24 hours, NF-κB activity in OFs from control individuals (#71, #116, #136) and patients with TAO (#120, #135, #137) was determined by measuring the levels of degradation of IκB and phosphorylation of IKKα/β and NF-κB p65 via a Western blot analysis. (B) OFs from control individuals (#111, #152, #155, 158) and patients with TAO (#107, #110, #135, #162) were co-transfected with pGL4.32[luc2P/NF-κB-RE/Hygro] for firefly luciferase and pRL-TK for renilla luciferase. After 24 hours, cell extracts were assayed for firefly and renilla luciferase activity. The results are expressed as the ratio of firefly-to-renilla luciferase activity (Fluc/Rluc).

OFs from patients with TAO (#120, #135, #137) showed higher levels of IKKα/β and p65 phosphorylation and a higher level of IκB degradation (Fig 4A), compared to controls (#71, #116, #136). We examined the activity of NF-κB-dependent promoters that contain binding sites for NF-κB. OFs were co-transfected with pGL4.32(luc2P/NF-κB-RE/Hygro) for Firefly luciferase and pRL-TK for Renilla luciferase. Consistent with the activity of the NF-κB pathway (Fig 4A), higher levels of luciferase activity were observed in OFs from patients with TAO (#107, #110, #135, #162), compared to controls (#111, #152, #155, #158) (Fig 4B). These findings indicate that OFs from TAO patients have a higher activity of the NF-κB pathway.

### Treatment with Bay 11–7082, an inhibitor of the NF-κB pathway, reduced the levels of IGF-1 secretion and the expression of *IGF-1* mRNA in OFs from patients with TAO

**Fig 5:**
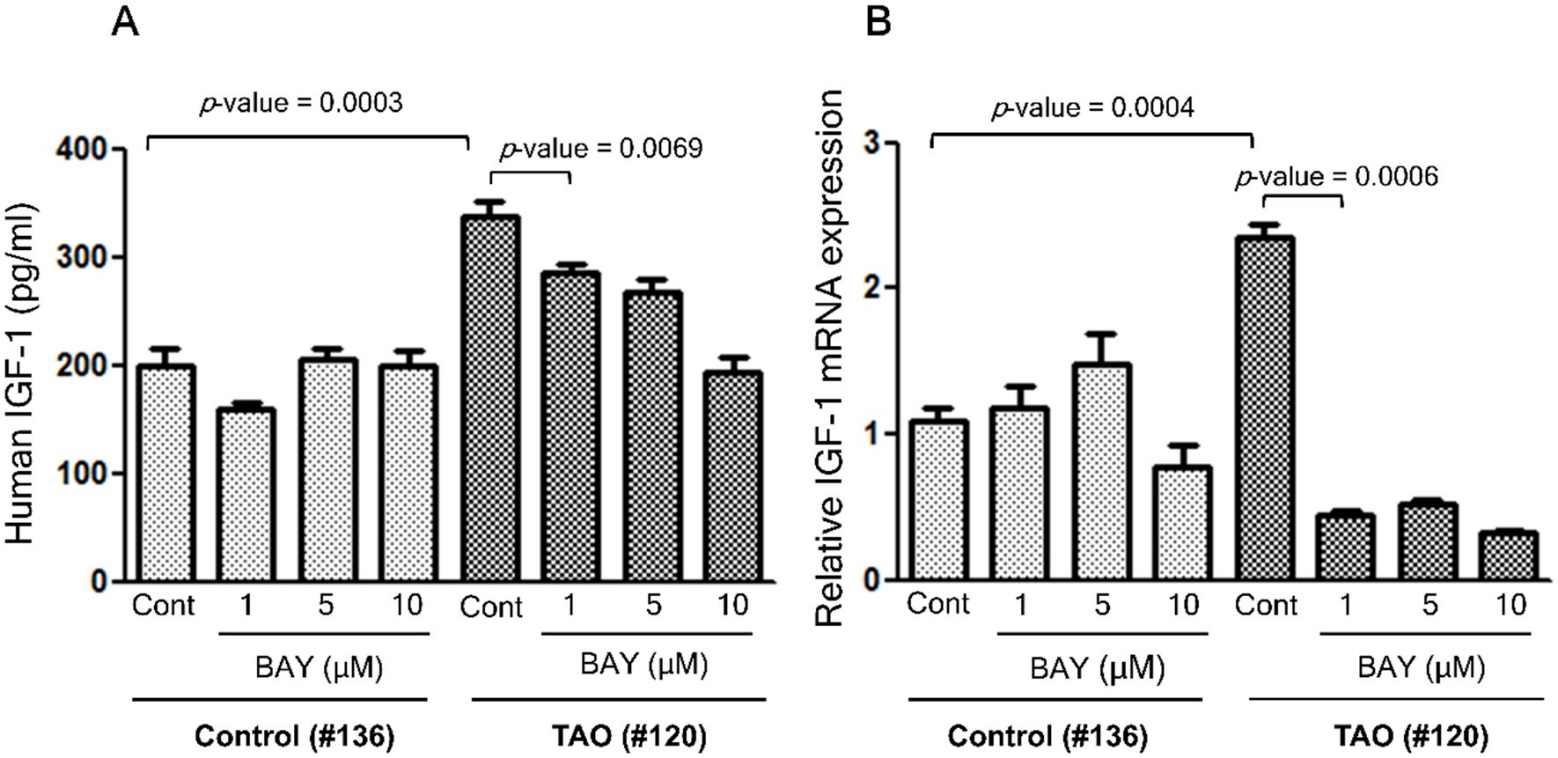
Treatment with Bay 11–7082, an inhibitor of the NF-κB pathway, reduced the levels of IGF-1 secretion and expression of *IGF-1* mRNA in OFs from patients with TAO. (A) OFs from one control individual (#136) and one patient with TAO (#120) were plated at a concentration of 2 x 10^5^ cells/well in a 6-well plate. After 24 hours, cells were treated with Bay 11–7082 at the indicated concentration for 24 hours. The culture medium was analyzed for IGF-1 content with ELISA. (B) *IGF-1* mRNA expression was determined by quantitative real time-RT–PCR.

We investigated whether the higher level of IGF-1 expression is due to the higher activity of NF-κB pathway. We investigated the effect of Bay 11–7082, an inhibitor of the NF-κB pathway, on IGF-1 secretion and *IGF-1* mRNA expression. Treatment with Bay 11–7082 reduced the level of IGF-1 secretion (Fig 5A) and expression of *IGF-1* mRNA (Fig 5B) in OFs from a patient with TAO (#120), whereas it had no effect on a control individual (#136), suggesting that the higher level of IGF-1 is associated with a higher activity of the NF-κB pathway in TAO patients.

### Treatment with octreotide reduced NF-κB activity in OFs from patients with TAO

**Fig 6:**
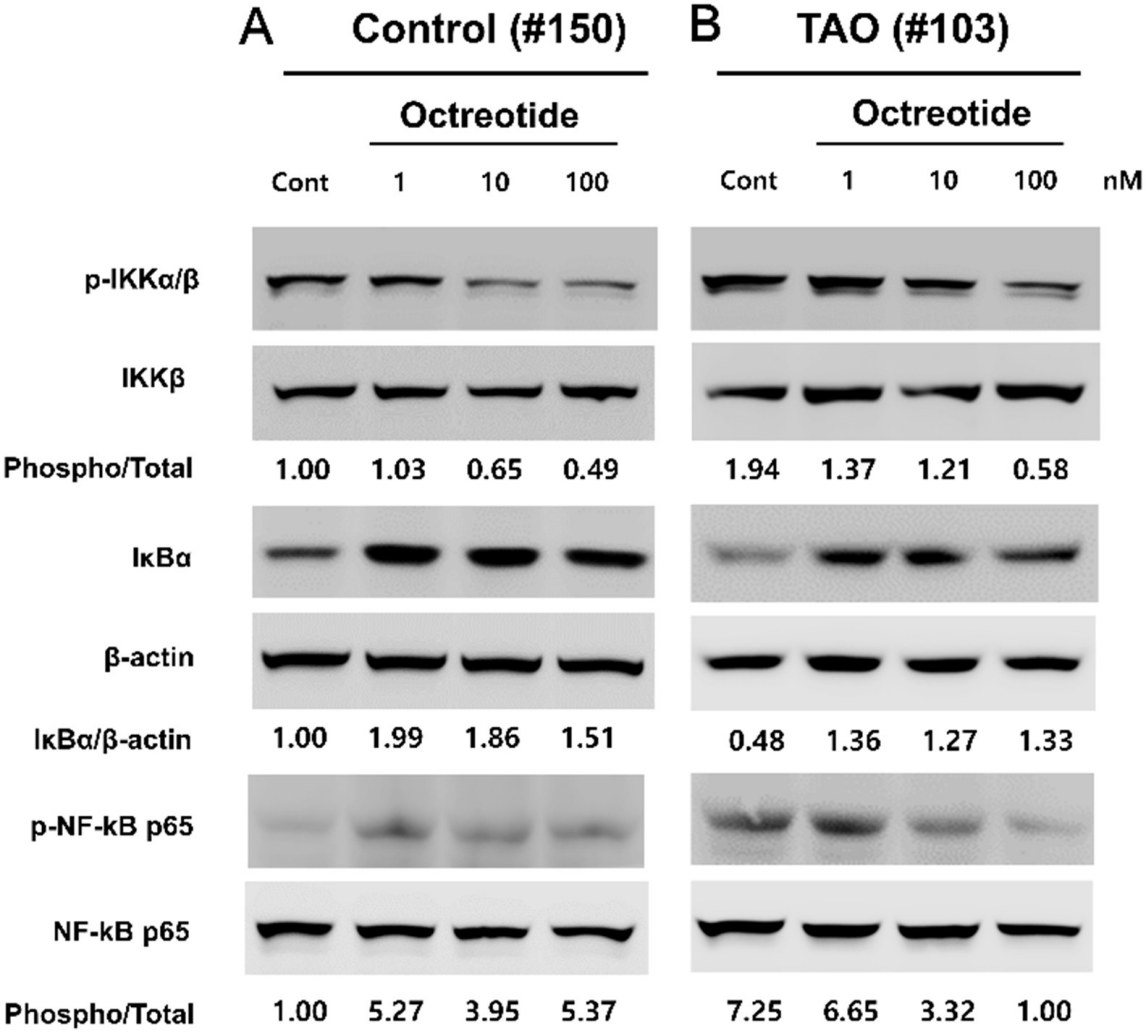
Treatment with octreotide reduced NF-kB activity in OFs from patients with TAO. OFs from a control individual (#150) and a patient with TAO (#103) were plated at a concentration of 2 x 10^5^ cells/well in a 6-well plate. After 24 hours, NF-κB activity in OFs was determined by measuring the levels of IκB degradation, and IKKα/β and NF-κB p65 phosphorylation via Western blot analysis.

We examined whether octreotide reduces NF-κB activity in OFs. Consistent with the results shown in Fig 4, non-treated OFs from patients with TAO showed higher levels of IKKα/β and p65 phosphorylation, and a higher level of IκB degradation, compared to controls (Fig 6). Treatment with octreotide inhibited phosphorylation of IKKα/β and p65, and degradation of IκB in OFs from a patient with TAO (# 103) (Fig 6B). Interestingly, in OFs from a control individual (#150), treatment with octreotide also inhibited phosphorylation of IKKα/β and degradation of IκB, whereas it did not affect phosphorylation of p65 (Fig 6A). These results indicate that octreotide inhibits IGF-1 secretion through the reduction of NF-κB activity in TAO patients.

## Discussion

The findings reported here indicated that treatment with octreotide, an SST analogue, inhibits secretion of IGF-1 via downregulation of the NF-κB pathway in OFs from patients with TAO.

In this study, to enhance the reliability of the experimental results, we used cells between passages 5 to 10 and cells did not exceed passage 10. The patients with TAO had experienced at least six months in inactive disease status under euthyroid hormonal conditions. Since the number of cells that can be obtained from a patient is limited, it was not possible to use OFs derived from the same patient for all experiments.

Although variations exist between OFs from different patients with TAO in the levels of IGF-1 secretion, SSTR2 expression, and NF-κB activity, OFs from patients with TAO consistently showed higher levels of IGF-1 secretion, SSTR2 expression, and NF-κB activity compared to controls, in the basal status without any treatment, even after five passages in vitro after separation from orbital tissues. We, thus, focused on the effect of octreotide on the baseline level of NF-κB activity in unmanipulated OFs.

Consistent with higher IGF-1 level in TAO patients, the level of IGF-1 secretion by cultured OFs from TAO patients has been reported to be higher than that from controls and was positively correlated with clinical activity score (CAS) in TAO patients [5]. The IGF-1/IGF-1 receptor (IGF-1R) pathway is now considered as a promising therapeutic target for TAO treatment, because OFs express IGF-1R, which potentiates TSHR signaling via formation of a functional complex with TSHR [22]. In addition, our previous study showed that IGF-1 enhanced cell surface functional TSHR levels, not only by increasing TSHR expression, but also by inducing TSHR translocation to the plasma membrane in OFs from TAO patients [23]. Moreover, in two clinical trials for active moderate-to-severe TAO, Teprotumumab, a human monoclonal antibody against IGF-1R, has demonstrated substantial and rapid improvement in clinical activity score and proptosis reduction in patients with TAO compared to placebo treatment [24]. These results indicate that the IGF-1/IGF-1R pathway plays an important role in the development of TAO.

In addition to IGF-1, the expression level of SSTR is also increased in OFs from TAO patients, compared to controls. Cozma et al. [8] reported that the levels of *SSTR1* and *SSTR2* mRNA expression were higher in OFs from patients with TAO than those from controls, although the increase in *SSTR2* mRNA expression was not statistically significant. The expression levels of SSTRs are also increased in lymphocytes derived from orbital tissues of patients with TAO. Pasquali et al. [9] reported that mRNAs of all *SSTR* family members were detected in lymphocytes from TAO retroorbital tissues and also in cultured lymphocytes from orbital tissue from TAO patients. In that report, the authors also showed that *SSTR1*, *SSTR2*, and *SSTR4* mRNA expression was higher than *SSTR3* and *SSTR5* expression in lymphocytes from patients with TAO, but only *SSTR2*, *SSTR3*, and *SSTR4* mRNAs were detectable at low level in lymphocytes from retroorbital tissues of control individuals.

NF-κB, a transcription factor, is ubiquitously expressed in most cells and is involved in various biologic responses, such as cell growth and survival, inflammation, immunity and oncogenesis [25]. In the current study, we investigated whether octreotide inhibits secretion of IGF-1 via suppression of the NF-κB pathway in OFs from patients with TAO. Our results show that OFs in TAO maintained higher activity of the NF-κB pathway (Fig 4), expression of IGF-1 was dependent on the NF-κB pathway (Fig 5), and that octreotide inhibited phosphorylation of IKKα/β and p65, and degradation of IκB in OFs from TAO patients (Fig 6). Consistent with our results, previous studies reported that treatment with SST or its analogues suppressed oxidative stress and inflammatory reaction via downregulation of the NF-κB pathway in various cells, both in vitro and in vivo. Treatment with octreotide protected brain [26] and retina [27] through inhibition of oxidative stress and the NF-κB pathway in a mouse model of ischemia-reperfusion injury. Bai et al. [28] reported that treatment with SST decreased inflammatory reaction and oxidative stress via suppression of the NF-κB pathway in rat microglial cells.

Although SST exhibits a variety of cell signaling depending on the subtype of receptor (SSTR1-5) SST binds to, both activation of phosphotyrosine phosphatases (PTPs) and inhibition of adenylyl cyclase (AC) are induced by all SSTR subtypes [29]. SSTR signaling seems to activate or inhibit the NF-κB pathway while cross-talking with the NF-κB pathway at various levels. In line with this, the effect of cAMP accumulation on the NF-κB pathway appears to vary according to the cell type and the conditions under study. The effect of cAMP accumulation by forskolin, an activator of AC, on the NF-κB pathway is stimulatory in 3T3 fibroblasts [30], whereas it is inhibitory in CHO cells [31]. In case of OFs from TAO patients, Pasquali et al. [20] reported that treatment with octreotide significantly decreased foskolin-induced cAMP accumulation, inhibited cell growth, and induced apoptosis.

How octreotide inhibits the NF-κB pathway is presently unknown, but the inhibition of AC by octreotide-induced SSTR activation is likely to participate in this process. Because the inhibition of AC by octreotide leads to the lowering of cAMP level in OFs [20] and the accumulation of cAMP is known to activate the NF-κB pathway in 3T3 fibroblasts [30], we reasoned that SSTR-induced lowering of cAMP may provide a link between octreotide and downregulation of the NF-κB pathway.

Our results show that treatment with Bay 11–7082, an inhibitor of the NF-κB pathway, reduced expression of IGF-1 in OFs from patients with TAO (Fig 5). Although the role of the NF-κB pathway is well characterized in downstream signaling of growth hormone (GH)/IGF-1 pathway, less is known about whether/how NF-κB is involved in the expression of IGF-1. As far as we know, STAT5b is the only known transcription factor, which is involved in the expression of *IGF-1* gene [32]. It has been reported that GH induces IGF-1 synthesis via activation of the transcription factor STAT5b in liver [33]. A research by Wu et al. [19] supports our results. They reported a possibility that, along with STAT5b, the NF-κB pathway could also be involved in the expression of *IGF-1* gene. They showed that the silencing of STAT5b prevented the GH-induced NF-κB p65-DNA binding and vice versa. The inhibition of NF-κB p65 also prevented the GH-induced STAT5b phosphorylation, supporting a reciprocal functional interaction between NF-κB p65 and STAT5b in GH-induced IGF-1 expression.

Medically, treatment with corticosteroids is still the first therapeutic option for moderate-to-severe and threatening optic neuropathy of TAO. However, since corticosteroids present a high risk of side effects, including impaired glucose tolerance, hypertension, iatrogenic Cushing’s syndrome, other alternatives are being suggested for medical management of TAO patients [34]. SST analogues, octreotide and lanreotide, are also considered as alternative treatment options for TAO. However, clinical trials of octreotide long-acting repeatable (LAR), conducted in mid-2000s, did not show significant therapeutic effects in patients with moderately severe TAO [16–18]. The researchers performed double-blind, placebo-controlled trials for 16 or 32 weeks and evaluated the therapeutic effects of octreotide on the basis of changes of the severity index and clinical activity scales of TAO. Interestingly, Stan et al. [18] and Wemeau et al. [17] reported that although overall therapeutic efficacy of octreotide on moderate-to-severe TAO was disappointing in their 16-week clinical trials, treatment with octreotide significantly reduced lid retraction and proptosis, respectively. Dickson et al. [16] conducted a longer clinical trial (32 weeks) and evaluated 50 patients with moderately severe and active TAO. They showed that as time went on, scores of soft tissue inflammation and clinical activities of TAO were reduced in both octreotide LAR and placebo groups and no significant therapeutic effect of octreotide LAR was observed at the final point after 32 weeks. However, interestingly, during the first 16 weeks, there was a significant reduction in the ophthalmopathy index (OI), as determined by the clinical features of TAO, including soft tissue inflammation, proptosis, and diplopia, in the octreotide LAR group compared to the placebo group.

It is currently unclear why octreotide is effective in TAO patients initially (0–16 week), but not later (16–32 weeks); it may be due to the difference in SSTR expression between the early and late stages of octreotide administration. It is also possible that the effect of octreotide decreases as the level of SSTR decreases during octreotide treatment. The accumulation of radiolabeled SST analogue in retroorbital tissue has been demonstrated in vivo with ^111^In-DTPA-_D_-Phe^1^-octreotide (Octreoscan) scintigraphy [35–38] and the level of uptake of somatostatin analogs in orbital scintigrams was positively correlated with TAO activity, supporting the possibility that SSTR expression may decrease during treatment.

In conclusion, IGF-1 secretion in OFs from patients with TAO is increased via the up-regulation of NF-κB activity and octreotide reduces NF-κB activity leading to a decrease in IGF-1 secretion. While treatment with octreotide may not be warranted in TAO patients, it may be beneficial for patients with high SSTR2 levels.

## Supporting information

S1 Fig IGF-1 ELISA. (XLSX)

S2 Fig *IGF-1* mRNA RT-PCR. (XLSX)

S3 Fig IGF-1 ELISA with treatment with octreotide. (XLSX)

S4 Fig Octreotide cytotoxicity. (XLSX)

S5 Fig SSTR2 flow cytometry. (PDF)

S6 Fig NK-κB promoter activity and luciferase assay. (XLSX)

S7 Fig IGF-1 ELISA with treatment with Bay 11–7082. (XLSX)

S8 Fig *IGF-1* mRNA RT-PCR with treatment with Bay 11–7082. (XLSX)

S1 Raw images Blot/Gel original data. (PDF)
