# Supplementary material for: Octreotide inhibits secretion of IGF-1 from orbital fibroblasts in patients with thyroid-associated ophthalmopathy via inhibition of the NF-κB pathway
Source: PLoS One. 2021 Apr 22;16(4):e0249988. doi: 10.1371/journal.pone.0249988 (PMC8062018; doi:10.1371/journal.pone.0249988)

YSW 111

BD FACSDiva 8.0.1

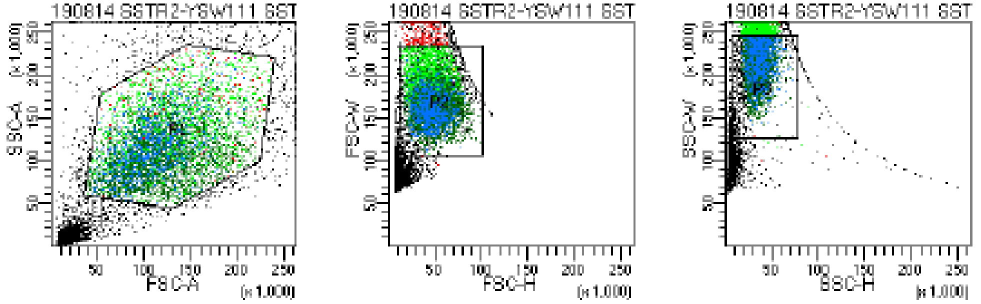

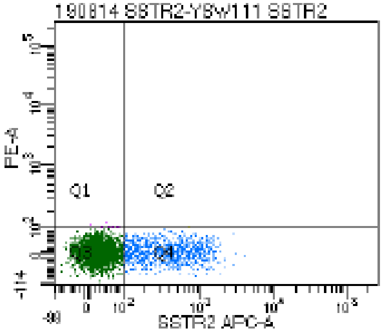

Tube: YSW111 SSTR2

Population	#Events	%Parent	%Total
All Events	10,000	####	100.0
P1	6,354	63.5	63.5
P2	6,076	95.6	60.8
P3	3,922	64.5	39.2
Q1	8	0.2	0.1
Q2	1	0.0	0.0
Q3	2,944	75.1	29.4
Q4	969	24.7	9.7

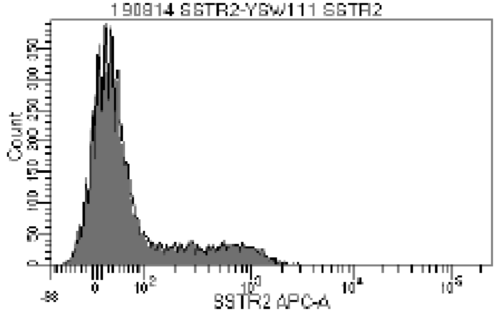

YSW 116

BD FACSDiva 8.0.1

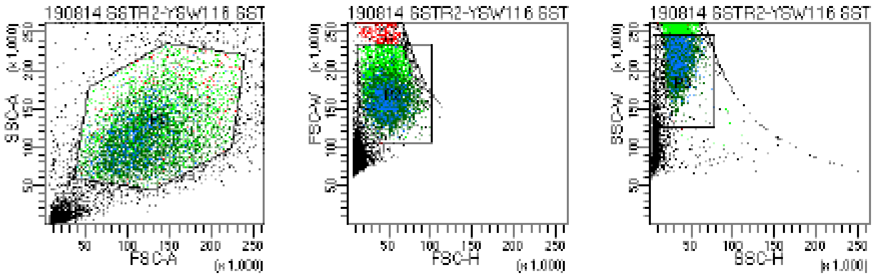

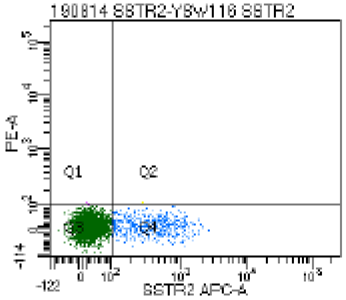

Tube: YSW116 SSTR2

Population	#Events	%Parent	%Total
All Events	10,000	####	100.0
P1	6,211	62.1	62.1
P2	5,917	95.3	59.2
P3	3,819	64.5	38.2
Q1	2	0.1	0.0
Q2	1	0.0	0.0
Q3	3,244	84.9	32.4
Q4	572	15.0	5.7

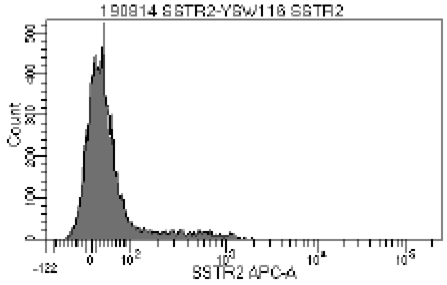

YSW 155

BD FACSDiva 8.0.1

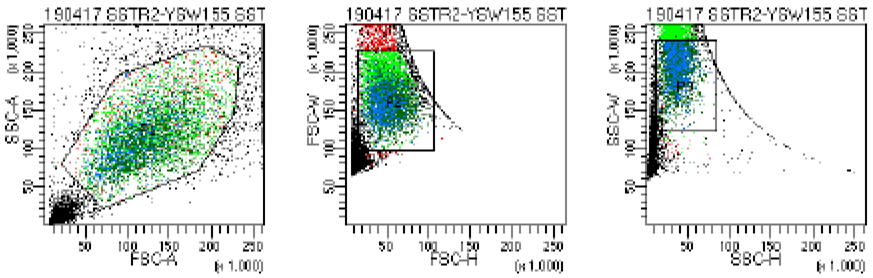

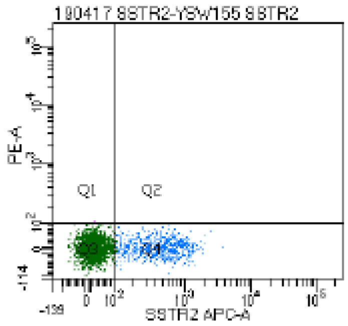

Tube: YSW155 SSTR2

Population	#Events	%Parent	%Total
All Events	10,000	####	100.0
P1	5,426	54.3	54.3
P2	4,938	91.0	49.4
P3	3,086	62.5	30.9
Q1	1	0.0	0.0
Q2	0	0.0	0.0
Q3	2,392	77.5	23.9
Q4	693	22.5	6.9

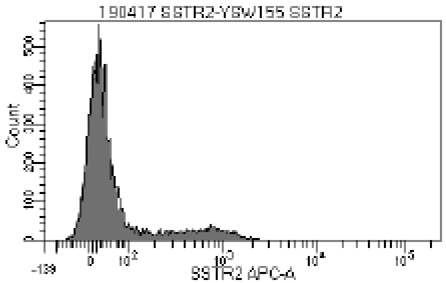

YSW 135

BD FACSDiva 8.0.1

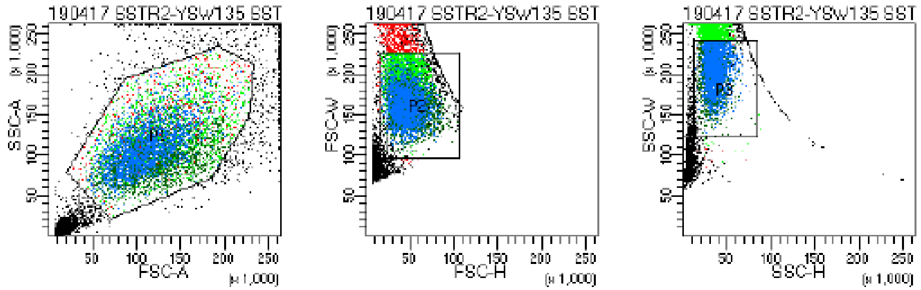

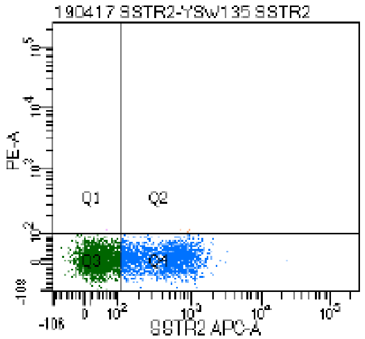

Tube: YSW135 SSTR2

Population	#Events	%Parent	%Total
All Events	10,000	####	100.0
P1	7,300	73.0	73.0
P2	6,668	91.3	66.7
P3	4,774	71.6	47.7
Q1	1	0.0	0.0
Q2	3	0.1	0.0
Q3	2,464	51.6	24.6
Q4	2,306	48.3	23.1

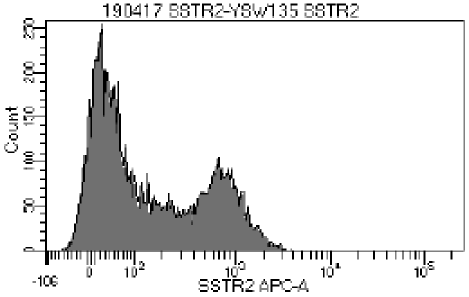

YSW 137

BD FACSDiva 8.0.1

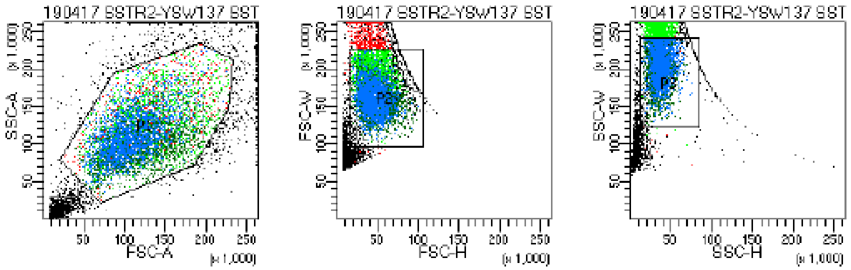

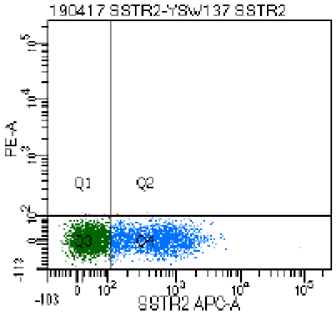

Tube: YSW137 SSTR2

Population	#Events	%Parent	%Total
All Events	10,000	####	100.0
P1	7,076	70.8	70.8
P2	6,461	91.3	64.6
P3	4,692	72.6	46.9
Q1	4	0.1	0.0
Q2	4	0.1	0.0
Q3	2,408	51.3	24.1
Q4	2,276	48.5	22.8

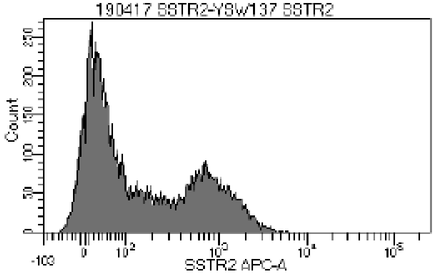

YSW 162

BD FACSDiva 8.0.1

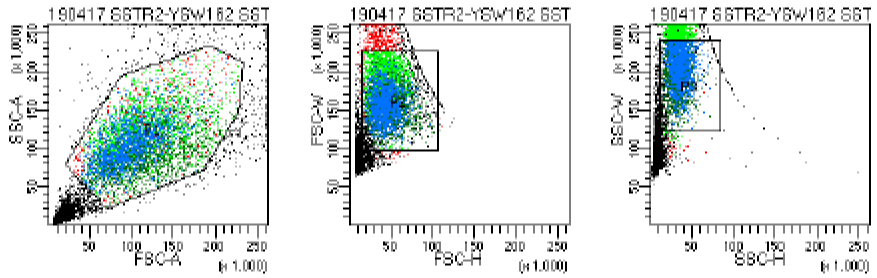

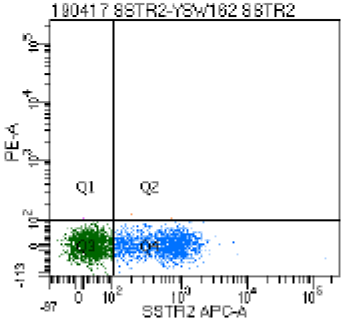

Tube: YSW162 SSTR2

Population	#Events	%Parent	%Total
All Events	10,000	####	100.0
P1	6,562	65.6	65.6
P2	6,069	92.5	60.7
P3	3,771	62.1	37.7
Q1	1	0.0	0.0
Q2	2	0.1	0.0
Q3	1,913	50.7	19.1
Q4	1,855	49.2	18.6

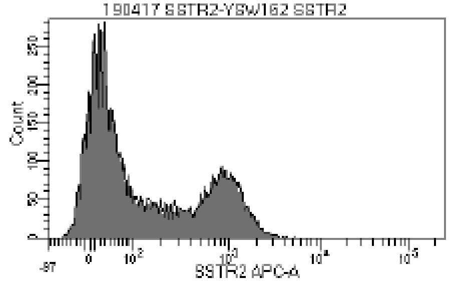

Supplement: S5 Fig — (PDF) [file pone.0249988.s005.pdf]
