# Supplementary material for: Octreotide inhibits secretion of IGF-1 from orbital fibroblasts in patients with thyroid-associated ophthalmopathy via inhibition of the NF-κB pathway
Source: PLoS One. 2021 Apr 22;16(4):e0249988. doi: 10.1371/journal.pone.0249988 (PMC8062018; doi:10.1371/journal.pone.0249988)
Supplement: S1 Raw images — (PDF) [file pone.0249988.s009.pdf]

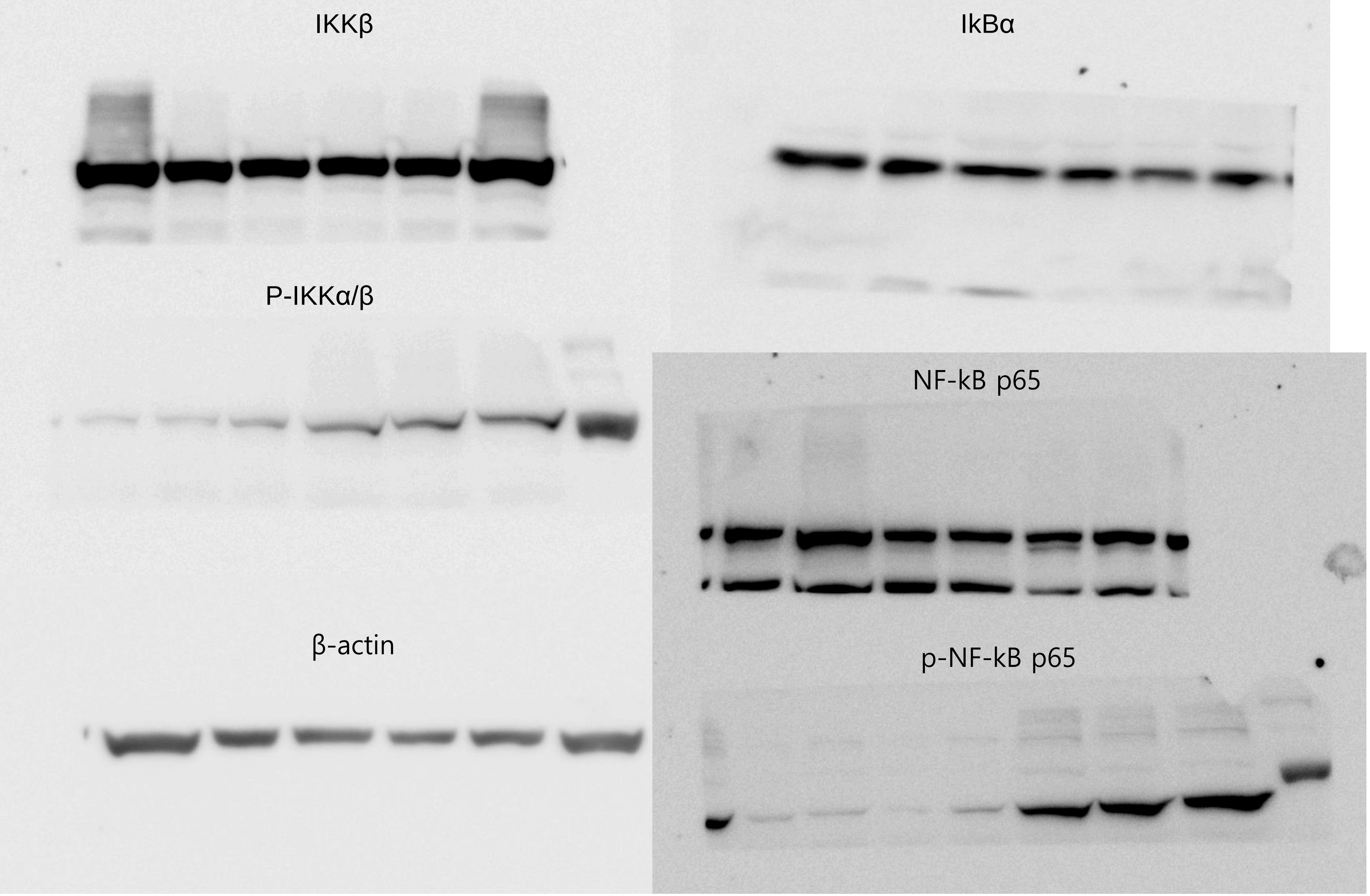
IKKβ
IkBα
P-IKKα/β
NF-kB p65
β-actin
p-NF-kB p65

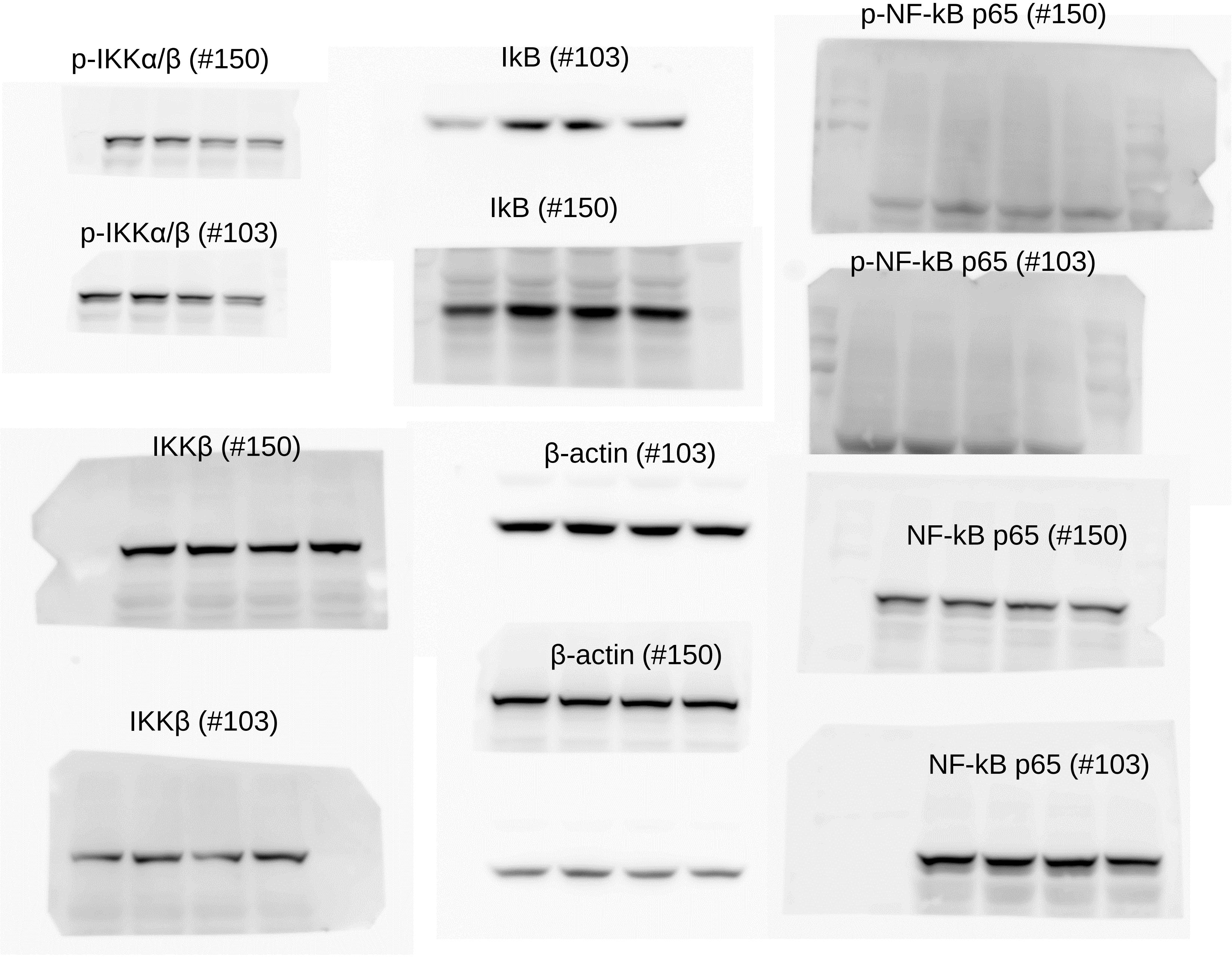
p-IKKα/β (#150)
p-IKKα/β (#103)
IkB (#103)
IkB (#150)
p-NF-kB p65 (#150)
p-NF-kB p65 (#103)
IKKβ (#150)
IKKβ (#103)
β-actin (#103)
β-actin (#150)
NF-kB p65 (#150)
NF-kB p65 (#103)
